# Bilirubin Triggers Calcium Elevations and Dysregulates Giant Depolarizing Potentials During Rat Hippocampus Maturation

**DOI:** 10.3390/cells14030172

**Published:** 2025-01-23

**Authors:** Giada Cellot, Giuseppe Di Mauro, Chiara Ricci, Claudio Tiribelli, Cristina Bellarosa, Laura Ballerini

**Affiliations:** 1 International School for Advanced Studies (SISSA), Neuroscience Area, Via Bonomea, 265, 34136 Trieste, Italy; gdimauro@sissa.it (G.D.M.); cricci@sissa.it (C.R.); 2 Fondazione Italiana Fegato ONLUS—Italian Liver Foundation, Bldg Q-AREA Science Park Basovizza, SS14 Km 163.5, 34149 Trieste, Italy; ctliver@fegato.it

**Keywords:** neuroscience, kernicterus, bilirubin neurotoxicity, calcium imaging, synaptic activity, patch clamp, Gunn rats

## Abstract

Neonatal hyperbilirubinemia may result in long-lasting motor, auditory and learning impairments. The mechanisms responsible for the localization of unconjugated bilirubin (UCB) to specific brain areas as well as those involved in potentially permanent central nervous system (CNS) dysfunctions are far from being clear. One area of investigation includes exploring how hyperbilirubinemia determines neuronal alterations predisposing to neurodevelopmental disorders. We focused on the hippocampus and pyramidal cell dysregulation of calcium homeostasis and synaptic activity, with a particular focus on early forms of correlated network activity, i.e., giant depolarizing potentials (GDPs), crucially involved in shaping mature synaptic networks. We performed live calcium imaging and patch clamp recordings from acute hippocampal slices isolated from wild-type rats exposed to exogenous high bilirubin concentration. We then explored the impact of endogenous bilirubin accumulation in hippocampal slices isolated from a genetic model of hyperbilirubinemia, i.e., Gunn rats. Our data show in both models an age-dependent dysregulation of calcium dynamics accompanied by severe alterations in GDPs, which were strongly reduced in hippocampal slices of hyperbilirubinemic rats, where the expression of GABAergic neurotransmission markers was also altered. We propose that hyperbilirubinemia damages neurons and affects the refinement of GABAergic synaptic circuitry during a critical period of hippocampal development.

## 1. Introduction

Prolonged and intense hyperbilirubinemia is characterized by increased levels of unconjugated bilirubin (UCB), which favors accumulation [1] into the CNS [2], to induce a severe chronic bilirubin encephalopathy named kernicterus. Kernicterus promotes irreversible neurological sequelae, including motor disorders, auditory dysfunctions and learning impairments [3,4,5], also depending on the CNS area undergoing bilirubin-free (Bf)-related neurodegeneration (i.e., cerebellum/basal ganglia, inferior colliculus or hippocampus [6]).

Bilirubin toxicity in the cerebellum and the inferior colliculus has been extensively studied (reviewed in [7,8,9]), while fewer works explored the effects of hyperbilirubinemia in the hippocampus. In vitro studies reported that a high dose of bilirubin, exogenously applied to the neuroblastoma cell line SH-SY5Y, dissociated primary hippocampal neurons or slices in culture triggered neuronal damage characterized by membrane leakage, reduced mitochondrial activity, endoplasmic reticulum (ER) stress, dysregulation of intracellular calcium homeostasis and apoptosis [10,11]. Yet, it is still underexplored whether bilirubin neuronal dysfunctional calcium homeostasis may be accompanied by synaptic alterations to affect complex network activity in the immature hippocampus, such as giant depolarizing potentials (GDPs, ref. [12]), whose emergence is critical for the reinforcement and consolidation of hippocampal synapses during early developmental stages [13].

In this work, we addressed the impact of bilirubin on pyramidal cells by combining calcium imaging and patch clamp recordings to monitor intracellular calcium dynamics and GDPs with single-cell resolution in acute hippocampal slices, an ex vivo preparation that preserves the cytoarchitecture of the tissue of origin [14]. We tested slices isolated from wild-type rats on different postnatal days, and we described age-dependent sensitivity to exogenously applied Bf at concentrations ranging between 40 and 140 nM. We observed that immature neurons (5–8 postnatal days) were more vulnerable to acute Bf exposures than older ones (9–10 postnatal), showing longer and synchronous alterations in calcium homeostasis and accelerated generation of GDPs.

To address the effects of endogenous bilirubin accumulation, we isolated acute hippocampal slices from the Gunn rat, a genetic model of hyperbilirubinemia emerged spontaneously in a Wistar rat colony [15]. These animals are a molecular and metabolic model of Crigler–Najjar syndrome type 1, which is characterized by lifelong unconjugated hyperbilirubinemia due to the lack of uridinediphosphoglucuronate glucuronosyltransferase-1 (UGT1A1)-mediated bilirubin glucuronidation [16]. Live imaging of hyperbilirubinemic Gunn hippocampal neurons showed age-dependent calcium homeostasis dysregulation. In Gunn hippocampal slices, we report a strong reduction in GDPs together with several alterations in the expression of GABAergic transmission markers.

We propose that hyperbilirubinemia damages neurons and affects the refinement of GABAergic synaptic circuitry within a certain time window during hippocampal postnatal development, leading to less robust GDP activity in hyperbilirubinemic animals.

## 2. Materials and Methods

### 2.1. Acute Hippocampal Slices

Transverse hippocampal slices were obtained from animals aged from 5 to 10 postnatal days (P5–P10), following the protocol described in [17], with minor modifications. We used Wistar rats and Gunn rats, which were heterozygous (Nj) or homozygous (jj) for a mutation in uridine diphosphate-glucuronylsyl transferase 1 (UGT1) [17,18]. The rats were obtained from the SPF animal facility of the University of Trieste (Bld Q2-AREA Science Park, Basovizza, Italy). All experiments were performed in accordance with EU guidelines (2010/63/UE) and Italian law (Decree 26/14) and approved by the local veterinary service, the animal wellbeing committee (OPBA) of the University of Trieste and the Italian Ministry of Health (1FF80.N.REL). The rats were decapitated, and their brains were removed from their skulls and placed into ice-cold dissection solution containing (in mM) 2 CaCl_2_, 3.5 KCl, 1.2 NaH_2_PO_4_, 1.3 MgCl_2_, 215 sucrose, 25 D-glucose, 25 NaHCO_3_, 0.0004 ascorbic acid, 1 kynurenic acid, 0.05 APV saturated with 95% O_2_ and 5% CO_2_, pH 7.3–7.4. Each brain was hemisected by a midline sagittal cut and stuck on a metal plate to allow slicing. Then, 300–350 μm thick slices were cut in dissection solution by using a Leica Vibratome (VT1000s). Afterward, the slices were transferred to a holding bath containing artificial cerebrospinal fluid (ACSF) saturated with 95% O_2_ and 5% CO_2_ and composed as follows (in mM): 2 CaCl_2_, 3.5 KCl, 1.2 NaH_2_PO_4_, 1.3 MgCl_2_, 130 NaCl, 25 D- glucose and 25 NaHCO_3_. The slices were left to recover for at least half an hour before calcium imaging or electrophysiological recordings.

### 2.2. Treatments

In blood, bilirubin is mainly bound to the carrier protein albumin, while only a small fraction results unbound, known as Bf. Three different Bf concentrations were tested as follows: 40 nM, 90 nM and 140 nM. Bilirubin (Sigma Aldrich, Waltham, MA, USA) was purified as described [18], dissolved in DMSO (3 μg/μL) and added to ACSF in the presence of BSA 30 μM. The concentrations of total UCB necessary to reach the desired Bf concentrations (40 nM, 90 nM and 140 nM) were calculated according to [19], amounting to, respectively, 7 μM, 14 μM and 21 μM in the presence of BSA 30 μM. To avoid bubble formation, UCB+BSA solution was prepared as 10X and diluted to 1X with 95% O_2_ and 5% CO_2_-saturated ACSF immediately before perfusion.

### 2.3. Live Calcium Imaging and Analysis

**Figure 1:**
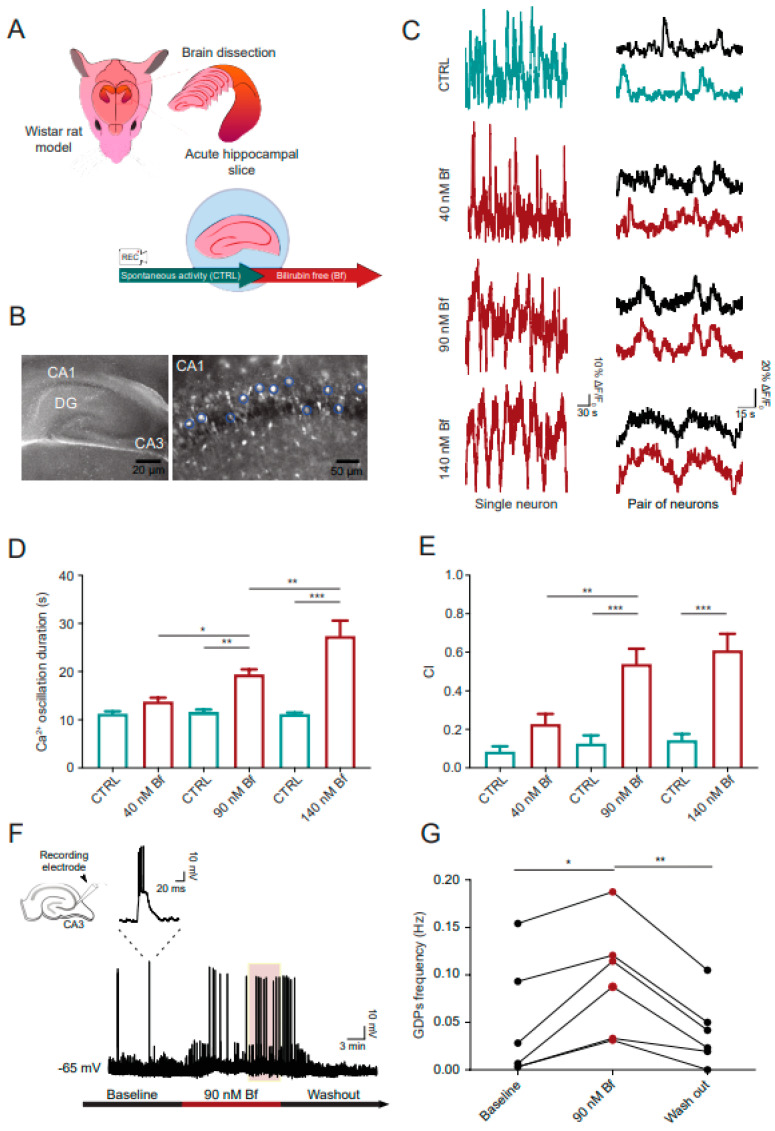
Exogenous bilirubin induces dose-dependent alterations in intracellular calcium signaling in ex vivo hippocampus and dysregulation of neuronal network emerging electrical activity. (**A**) Schematic representation of acute hippocampal slices obtained from wild-type rats for recording intracellular calcium activity before and after treatment with unconjugated bilirubin. (**B**) Representative fields of a hippocampal acute slice loaded with Fluo-4 AM fluorescent Ca^2+^ dye. On the left, an image showing the entire hippocampus: CA1-2-3, *Cornu Ammonis* 1-2-3; DG, Dentate Gyrus. On the right, a magnification of the CA1 region during calcium imaging recordings. Exemplative ROIs used for the analysis of neuronal cells labeled with blue circles. (**C**) Representative fluorescent traces of Ca^2+^ activity of CA1 single neurons (**left** column) and in pairs of neurons (**right** column, black tracings correspond to a second cell within the same filed) in spontaneous activity (CTRL, green traces, before treatment) and in Bf treatments (40 nM, 90 nM, 140 nM, red traces). (**D**) Bar plot of Ca^2+^ oscillation duration using different doses of Bf and (**E**) of the CI, measuring the synchronization of Ca^2+^ oscillations from pairs of neurons in the same optical field. For both, statistical significance by two-way ANOVA, with Sidak’s multiple comparison test; N = 8 samples for the dose at 40 nM and 90 nM Bf and N = 9 samples for the dose 140 nM Bf. (**F**) Patch clamp current clamp recording of GDPs in acute hippocampal slices. Left top inset: schematic representation of a hippocampal acute slice with a patch pipette targeting the CA3 region. After a 10 min recording of baseline activity, 90 nM Bf was applied for 15 min (red bar below the trace). The light red area superimposed on the trace represents the time window considered for analyzing Bf-induced changes in GDPs (**G**). Plot showing the changes in GDP frequency during baseline, Bf application and washout. Statistical significance by one-way ANOVA, with Tukey’s post hoc test; N = 6 slices from 5 animals. Dots interconnected by lines are values recorded from the same cell. Statistical differences are reported as * *p* < 0.05, ** *p* < 0.01, *** *p* < 0.001.

The monitoring of intracellular calcium signals in acute hippocampal slices was performed by using a 4 μM Fluo-4 AM (Invitrogen, London, UK) fluorescent indicator. Slices were loaded with the dye by incubation in the dark for 30 min in 95% O_2_ and 5% CO_2_-saturated ACSF at 37 °C; then, they were moved to a recording chamber placed on an inverted microscope (Nikon Eclipse Ti-U) and perfused at a rate of 3 mL/minute with saturated ACSF solution alone or with treatments. Recordings were performed at 34 °C. To prevent the samples from movement while perfused, each slice was kept in position through a homemade U-shaped slice anchor. The slices were recorded with a 20× objective (0.45 NA). This magnification enabled to focus on the region of interest (the CA1 hippocampal area) and on the cells (Figure 1A). The excitation light (488 nm) was provided by a mercury lamp (Nikon intensilight C-HGFI) and separated from the emitted one through a 395 nm dichroic mirror and ND filter (1/8). The live imaging recordings were performed by acquiring continuous images at 150 ms exposure time, binning 4 × 4 and resolution 512 × 512 by means of a Hamamatsu sCMOS camera (C11440-22CU ORCA-Flash4.0). The recording system was controlled by HCImage Live imaging software, version 1.0.

In acute slices obtained from wild-type Wistar animals, calcium spontaneous activity was recorded in ACSF solution (≥10 min) prior to treatments. Then, the samples were perfused for 15 min with Bf, and calcium oscillations were analyzed in the last 10 min of recordings. At the end of Bf treatment, the slices were recorded in the presence of tetrodotoxin (TTX, 1 μM, Hello Bio, Bristol, UK), an inhibitor of action potential generation, to confirm the neuronal nature of the recorded cell [20]. In some experiments, TTX was applied before Bf in order to study the contribution of synaptic activity to Bf-induced alterations in calcium dynamics. In experiments performed on Gunn rat acute slices, recordings were performed in ACSF, and TTX was post-applied to confirm the independence of large calcium events from synaptic activity in the selected cells. In the jj animals, 80% of the analyzed cells presented long calcium oscillations after TTX application (N = 318 cells), whose duration was not changed by TTX treatment (before and after TTX, 26.11 ± 2.73 s and 25.68 ± 2.11 s, respectively; N = 10 cells from three different jj animals).

For each set of experiments, at least 4 animals from different breeding were used.

The analysis was performed with Fiji software, version 1.53, using the Region Of Interest (ROI) selection tool (N = 10 ± 5 cells from each sample) to identify neuronal active cells in the *stratum pyramidale* of the CA1 region and extrapolate their fluorescent profiles. Fluorescence intensity was expressed as ΔF/F0, where ΔF corresponds to the rise over the baseline (F0).

Clampfit software, version 10, was used to analyze Ca^2+^ fluorescence recordings (≥10 min), quantifying the duration, frequency and synchronization of neuronal intracytoplasmic Ca^2+^ events. The duration of Ca^2+^ oscillations was defined as the time from the onset of the calcium rise over the baseline to the time when the amplitude declined back to 95% of the baseline. Only oscillations with an amplitude ten times larger than the standard deviation of the noise were considered. Frequencies were calculated as the number of oscillations divided by the recording time. Cell synchronization was evaluated in pairs of cells in the same optical field approximately 200 µm apart, using the cross-correlation index (CI), with CI = 0 and CI = 1 indicating complete desynchronization and synchronization, respectively.

In the control solutions, the duration of calcium oscillations was slightly longer in the Nj animals with respect to the control WT ones (at P5–6 *p* < 0.05, at P7–8 *p* > 0.05 and at P9–10 *p* < 0.0001).

### 2.4. Electrophysiological Recordings and Analysis

Electrophysiological activity was recorded from CA3 pyramidal neurons of acute slices perfused with ACSF, visually identified with an upright Nikon microscope equipped with differential interference contrast optics and an infrared video camera, by using a patch clamp amplifier (Multiclamp 700A, Axon Instruments, Whipple Rd Union City, CA, USA). Recordings were performed at 34 °C.

The whole-cell patch clamp technique was used in voltage and current clamp modes. Patch electrodes were pulled from borosilicate glass capillaries and had a resistance of 4–7 MΩ when filled with an intracellular solution containing (in mM) 120 K gluconate, 20 KCl, 10 HEPES, 10 EGTA, 2 MgCl_2_ and 2 Na_2_ATP (pH 7.3, osmolarity adjusted to 300 mOsm).

Data were transferred to a computer hard disk after digitization with an A/D converter (Digidata 1322, Molecular Devices, San Jose, CA, USA). Data acquisition (digitized at 20 kHz and filtered at 2 kHz) was performed with pClamp 10.7 software (Molecular Devices, USA).

GDPs were recorded in current clamp mode from cells held at −65 mV of membrane potential through current injection. In acute slices obtained from wild-type Wistar rats, after recording GDP activity for at least ten minutes, 90 nM Bf was applied for 15 min, and changes in GDPs were analyzed in the last 5 min of the recording. In slices obtained from Gunn rats, GDPs were recorded without any treatment.

Cell capacitance and input resistance were recorded in voltage clamp mode and measured online with the membrane test feature of pClamp software. Induced firing activity was evaluated in current clamp mode by injecting 100 ms long-lasting current steps of increasing amplitude (0, 20, 40, 60, 80, 100, 120, 140 pA) from a holding potential of −65 mV.

Spontaneous postsynaptic activity was recorded at a holding potential of −56 mV. For each trace, amplitude and inter-event intervals of GABAergic slow decaying (~15 ms) postsynaptic currents (PSCs) were evaluated offline with Axograph 1.4.4 event detection software, which exploits a detection algorithm based on sliding templates to collect synaptic events.

Values of membrane potential were corrected for a liquid junction potential of ~16 mV (calculated with Clampex software, version 10; Molecular Devices, USA). The stability of the patch was checked by repetitively monitoring the input and series resistance during the experiments. Cells exhibiting 15% changes were excluded from the analysis. The series resistance, <20 MΩ, was not compensated.

### 2.5. Western Blot Analysis

Whole hippocampi were dissected from P5 and P10 Nj and jj Gunn rats. Protein isolation from a single hippocampus was performed using RIPA buffer (150 mM NaCl, 1% NP-40, 0.5% DOC, 0.1% SDS, 50 mM Tris pH 8, 1 mM protease inhibitor cocktail) added in tissue softened on ice. The samples were triturated using a 1000 μL pipette and 1 mL syringe pass tissue suspension through a 25-gauge needle until all tissues were lysed. The samples were agitated in an end-over-end rotator for 1 h at 4 °C and then sonicated at 25% amplitude for 5 s three times before being centrifugated at 10,000 rpm for 30 min (4 °C). The supernatants were transferred to a new pre-chilled tube, and the pellets were discarded. The protein concentration of the single hippocampus lysate was determined using the Bradford assay (Thermofisher, Waltham, MA, USA). Subsequently, the samples were prepared by adding Leammli buffer 2× (10% SDS, 20% glycerol, 125 mM Tris-HCl, 0.01% bromophenol blue, 1 M DTT) to 20 μg of proteins and denatured by boiling at 100 °C for 5 min. Separating SDS-PAGE gels were used at the concentration of 10% polyacrylamide. The samples were run and then transferred to an Immun-Blot PVDF membrane (Millipore, Burlington, MA, USA) by electroblotting. The membranes were blocked in 5% BSA in TBS-T for 1 h and incubated overnight at 4 °C with anti-NKCC1 (rabbit monoclonal, 1:1000, Abcam, Tokyo, Japan), anti-KCC2 (rabbit monoclonal, 1:1000, Abcam), anti-GAT-1 (rabbit polyclonal, 1:1000, Alomone, Jerusalem, Israel) and anti-GAPDH (mouse monoclonal, 1:000, Proteintech, Tokyo, Japan) for the housekeeping protein normalizer. The day after, the membranes were washed in TBS-T and incubated with a secondary antibody (Horseradish peroxidase-conjugated anti-rabbit or anti-mouse, 1:1000, Invitrogen, London, UK) diluted in blocking solution for 1 h at RT and washed again in TBS-T. The membranes were developed by enhanced chemiluminescence (ECL Western Blotting Substrate, Thermofisher) using the UVITEC Cambridge system. The quantification of the band fluorescent intensity was performed using Fiji software. Proteins of interest were expressed as fold change (FC) values normalized on GAPDH expression.

### 2.6. Statistics

In all the experiments, the Gaussian distribution of the datasets was assessed through the D’Agostino–Pearson omnibus normality test. If following Gaussian distribution, an ordinary one-way ANOVA test was used to measure statistically significant differences among conditions, followed by Tukey’s or Fisher’s LSD post hoc tests. In the case of two conditions, Student’s or Welch’s *t*-tests were used. Data that did not follow a normal distribution were processed using a nonparametric one-way ANOVA followed by Dunn’s post hoc tests. In the case of two conditions, the Mann–Whitney test was used. A two-way ANOVA followed by Sidak’s multiple comparison test was used when two factors (age and treatment/genotype) could affect the response. The statistical significance of differences in cell percentages presenting GDPs was assessed through the Chi-Squared test. Spearman’s correlation coefficients were measured to quantify the relation between calcium oscillation durations and CI. Differences between cumulative distributions were assessed through the Kolmogorov–Smirnov test. All the data were presented as Mean ± SE. The statistical significance was determined as follows: * *p* < 0.05, ** *p* < 0.01 and *** *p* < 0.001.

## 3. Results

### 3.1. Bilirubin Dose-Dependent Modulation of Neuronal Calcium Dynamics in Wild-Type Hippocampal Slices

We tested the impact of exogenous UCB on neuronal calcium dynamics in wild-type hippocampal slices (P5; Figure 1A). Acute slices were loaded with the membrane-permeable calcium-sensitive probe Fluo-4 AM (4 μM). Basal intracellular calcium variations were recorded from visually identified neurons in the *stratum pyramidale* (Figure 1B) for 10 min (control) prior to applying UCB (10 min) at increasing concentrations (Bf 40, 90 and 140 nM; [10]), with the lower dose being below and the higher dose being above the minimal amount reported to induce neurotoxicity (80 nM, [21]).

Figure 1C (left column) fluorescence tracings exemplify calcium dynamics in neurons (snapshot in Figure 1B) in the control and after Bf exposures. Control basal activity comprised an irregular slow intracellular calcium rise of 11.24 ± 0.52 s duration, which remained substantially unchanged by exposure to 40 nM Bf (13.82 ± 0.78 s in 40 nM Bf, recorded from N = 8 slices, *p* > 0.05; Figure 1C,D). Increasing UCB doses induced the appearance of significantly longer calcium events, whose duration was dose-dependent (19.43 ± 1.03 s at 90 nM Bf, N = 8 slices, *p* < 0.01) and increased to 27.39 ± 3.19 s in 140 nM Bf (N = 9 slices, *p* < 0.001; Figure 1C,D). The UCB effects on calcium event duration were significantly higher with increasing concentrations (40 nM Bf vs. 90 nM Bf: *p* < 0.05; 90 nM Bf vs. 140 nM Bf: *p* < 0.01, Figure 1D). The frequency of long calcium events due to Bf exposure was progressively reduced in accordance with the dose-dependent increase in these events’ duration (Supplementary Figure S1A).

In the control and UCB-treated samples, we quantified the extent of calcium event synchronization among different neurons by measuring the correlation index (CI) in pairs of simultaneously recorded cells (see methods; Figure 1C, right column). CI was low in basal activity and only slightly higher at 40 nM Bf (0.08 ± 0.02 CI in control and 0.22 ± 0.05 CI in 40 nM Bf, measured in N = 8 slices, *p* > 0.05). CI was instead significantly enhanced at 90 nM (0.12 ± 0.04 CI in the control and 0.53 ± 0.07 CI in 90 nM Bf, N = 8 slices, *p* < 0.001) and at 140 nM (0.14 ± 0.03 CI in the control and 0.61 ± 0.08 CI in 140 nM Bf, N = 9 slices, *p* < 0.001; Figure 1C–E). The synchronization of calcium events among different pyramidal cells increased significantly also when comparing 40 nM Bf to 90 nM Bf (*p* < 0.01) but not when comparing 90 nM Bf to 140 nM (*p* > 0.05, Figure 1E). To note, in Bf-treated neuronal pairs, when plotting calcium event duration against the corresponding CI, the Spearman’s correlation coefficient was close to zero, indicating that these two parameters were unrelated (Supplementary Figure S1B) and suggesting that the enhanced CI was not a mere consequence of calcium event prolongation.

Differently from the control calcium events, the long and synchronous calcium oscillations due to UCB exposures (Bf 90 nM and 140 nM) were independent of neuronal firing and synaptic activity, confirming previous observations obtained in cultured hippocampal neurons [10]. As shown in Supplementary Figure S1C,D, tetrodotoxin (TTX, 1 µM), a blocker of fast-inactivating voltage-gated sodium channels, removed all calcium events in the control, but when applying Bf (90 nM) in the presence of TTX, large and synchronized calcium events could still be induced (Supplementary Figure S1C–E).

### 3.2. In Wild-Type Hippocampal Slices, Bilirubin Transiently Dysregulates Network Activity Emerging During Early Postnatal Development: GDPs

In parallel experiments, we measured hippocampal network activity, focusing on GDPs, namely, collective, synchronized synaptic events built up by the concerted synergism of glutamatergic and GABAergic synapses. GDPs typically occur and shape hippocampal network development and synaptic maturation during the first postnatal weeks [12].

Figure 1F shows single-cell patch clamp recordings (current clamp mode) of GDPs from a pyramidal cell (P5–6) in the CA3 region, the area where GDPs are generated [22].

GDPs were recorded at a membrane potential of −65 mV (~0.025 nA negative current) and appeared as recurrent, suprathreshold membrane depolarizations, characterized by an underlying area of 12,770 ± 2226 mV*ms and a time to half maximum peak of 3.59 ± 0.51 ms (N = 6 slices; see the magnification in Figure 1F, inset), occurring spontaneously. After 10 min recording of this basal activity, we applied Bf (90 nM, 15 min), which induced an increment in GDP frequency (from 0.05 ± 0.02 Hz to 0.10 ± 0.02 Hz, *p* < 0.05; Figure 1F and plot in Figure 1G). This was associated with the absence of statistically significant changes in baseline membrane potential (−0.5 ± 1 mV) or in GDP area and time to half maximum peak (7631 ± 1622 ms*mV and 4.64 ± 1.59 ms). The increase in frequency was transient and reversed upon 10 min Bf washout, returning to baseline values (0.04 ± 0.01 Hz, *p* < 0.01 Bf vs. washout; Figure 1F,G), probably due to a progressive clearance of bilirubin following prolonged washout [10].

### 3.3. In Wild-Type Hippocampal Slices, Bilirubin Dysregulation of Intracellular Calcium Dynamics Depends on the Postnatal Age of the Hippocampus

**Figure 2:**
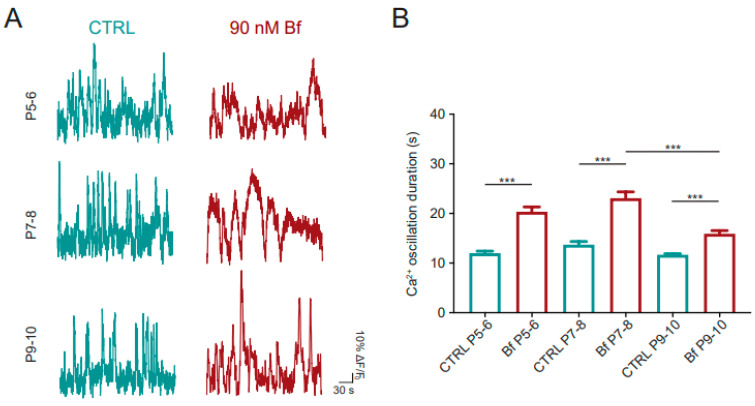
Bilirubin disrupts intracellular calcium dynamics of hippocampal neurons in an age-dependent manner. (**A**) Representative fluorescent traces of Ca^2+^ oscillations recorded from CA1 neurons before (CTRL, green traces) and after 90 nM Bf application (red traces) in acute slices obtained from animals at P5–6, P7–8 and P9–10. (**B**) Bar plot of Ca^2+^ oscillation duration in control and during Bf treatment at different developmental ages. Statistical significance by two-way ANOVA with Sidak’s multiple comparison test. N = 11 samples for each condition; statistical differences are reported as *** *p* < 0.001.

We used 90 nM Bf as the test dose to monitor calcium dynamics responses in neurons imaged from acute hippocampal slices isolated at three different postnatal ages, namely, P5–6, P7–8 and P9–10. In the presence of Bf, slow (see below) and long calcium events emerged in all age groups with respect to the controls (P5–6: from 12.02 ± 0.39 s in the control to 20.34 ± 0.97 s in Bf, *p* < 0.001; P7–8: from 13.72 ± 0.62 s in the control to 23.04 ± 1.27 s in Bf; *p* < 0.001; P9–10: from 11.67 ± 0.22 s in the control to 15.92 ± 0.64 s in Bf, N = 11 slices each age, *p* < 0.001; Figure 2A,B), but displaying an increase in the event duration that was significantly smaller in more mature hippocampi (Bf P7–8 vs. Bf P9–10: *p* < 0.001, Figure 2B). The lowest frequency of Bf calcium oscillations was measured at P7–8, in accordance with the age-dependent modulation of calcium event duration (Supplementary Figure S1F).

Similarly, the Bf-induced increase in the synchronization of calcium events appeared reduced in the older neurons, with CI values halved at P9–10 when compared to P7–8 (for P5–6: 0.11 ± 0.03 CI in the control and 0.51 ± 0.06 CI in Bf, *p* < 0.001; for P7–8: 0.08 ± 0.01 CI in the control and 0.47 ± 0.07 CI in Bf; *p* < 0.001; for P9–10: 0.05 ± 0.008 CI in the control and 0.25 ± 0.04 CI in Bf, *p* < 0.01; Bf P7–8 vs. Bf P9–10, *p* < 0.05).

Altogether, these results showed that from the beginning of the second postnatal week, the magnitude of calcium dynamics dysregulation due to Bf exposures progressively declines, suggesting that mature neurons are less vulnerable to Bf threat.

### 3.4. Hippocampal Neuron Intracellular Calcium Signaling Is Altered in an Age-Dependent Manner in Hyperbilirubinemic jj Gunn Rats

**Figure 3:**
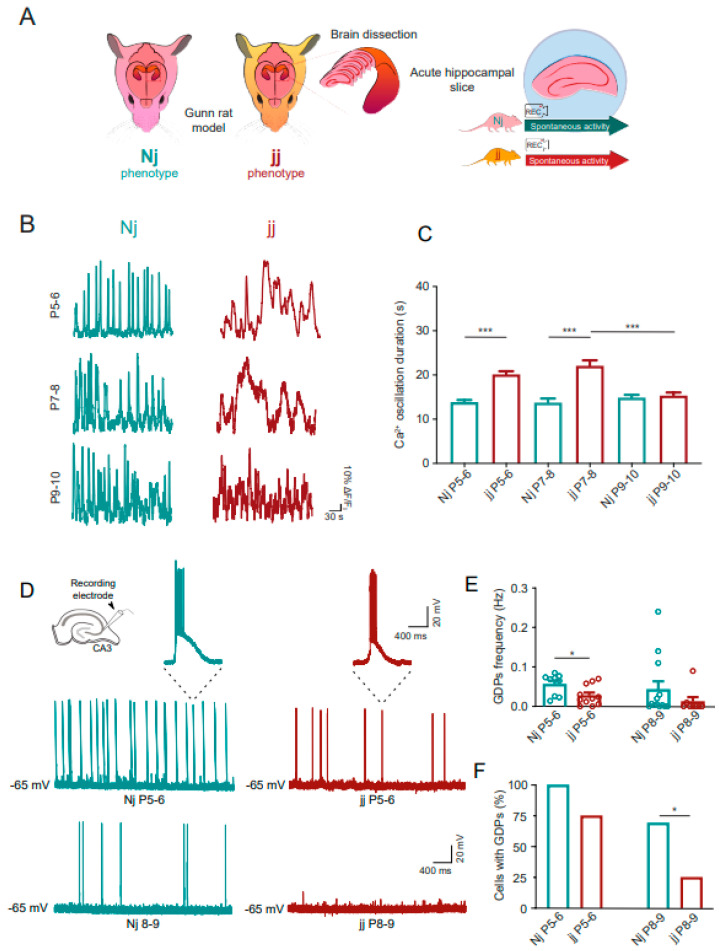
The hyperbilirubinemic Gunn rat (jj genotype) presents an age-dependent prolonged duration of intracellular calcium oscillations and dysregulation of GDPs with respect to the normobilirubinemic animal (Nj genotype). (**A**) Schematic representation of acute hippocampal slices obtained from normobilirubinemic Nj and hyperbilirubinemic jj rats for recording intracellular calcium activity. (**B**) Representative Ca^2+^ fluorescent traces obtained from CA1 neurons from heterozygous Nj (**left** column) and homozygous jj (**right** column) animals at different developmental ages. (**C**) Bar plot of spontaneous Ca^2+^ oscillation duration events. Statistical significance by two-way ANOVA with Sidak’s multiple comparison test; N = 11 samples for Nj P5–6, N = 13 for jj P5–7, N = 11 for Nj P7–8, N = 12 for jj P7–8, N = 10 for Nj P9–10 and N = 10 for jj P9–10. (**D**) Patch clamp recordings of GDPs in acute hippocampal slices from Nj (on the **left**) and jj (on the **right**) animals. Left top inset: schematic representation of a hippocampal acute slice with a patch pipette targeting the CA3 region. The top and bottom traces are recordings from P5–P6 and P8–P9 animals, respectively. In the magnifications on the top, single GDPs are shown for the Nj (**left**) and jj (**right**) genotypes. (**E**) Bar plot of GDP frequencies at different ages for the different genotypes. The dots superimposed on the bars are single-cell values. Statistical significance by Student’s *t*-test. At P5–6, for Nj, N = 9 slices from 4 animals, and for jj, N = 11 slices from 4 animals; at P8–9, for Nj, N = 13 slices from 6 animals, and for jj N = 8 slices from 5 animals. (**F**) Plot of the percentage of cells presenting GDPs at the different ages for the different genotypes. Statistical significance by the Chi-Squared test. Statistical differences are reported as * *p* < 0.05 and *** *p* < 0.001.

We explored the impact of chronically increased endogenous bilirubin during hippocampal development by exploiting live calcium imaging in ex vivo hippocampal slices isolated from the Gunn rats (Figure 3A). In detail, Gunn rats homozygous (jj) or heterozygous (Nj) for the genetic defect in UGT1A1 are hyperbilirubinemic and normobilirubinemic, respectively [16].

Live calcium imaging performed from neurons in CA1 *stratum pyramidale* in acute Gunn rat hippocampal slices isolated at three different ages (P5–6, P7–8 and P9–10; Figure 3B,C) revealed a dysregulation in calcium dynamics in early postnatal development when comparing hyperbilirubinemic jj to the normobilirubinemic control Nj; such a dysregulation disappeared during the second postnatal week.

In the jj genotype, in comparison to aged-matched Nj, the duration of spontaneous neuronal calcium elevations was significantly longer at both P5–6 (from 13.85 ± 0.48 s in Nj, N = 11 slices, to 20.14 ± 0.70 s in jj, N = 13 slices, *p* < 0.001) and P7–8 (from 13.74 ± 0.95 s in Nj, N = 11 slices, to 22.10 ± 1.21 s in jj, N = 12 slices, *p* < 0.001). Differently, no changes were found at P9–10 between the jj and Nj groups (from 14.89 ± 0.61 s in Nj, N = 10 slices, to 15.34 ± 0.70 s in jj, N = 10 slices, *p* > 0.05; Figure 3B,C), with more mature jj neurons displaying significantly shorter events (jj P7–8 vs. jj P9–10: *p* < 0.001, Figure 3C). The frequency of long calcium events in jj rats displayed the minimal value at P7–8 (Supplementary Figure S2A), in accordance with the postnatal age displaying the longer calcium oscillations.

The synchronization of calcium oscillations among neurons was also increased in jj with respect to Nj, but it was limited to the first week of postnatal development. This was measured through the CI, which was enhanced in jj at P5–P6 (0.16 ± 0.01 CI in Nj and 0.34 ± 0.03 CI in jj, *p* < 0.001) and at P7–8 (0.16 ± 0.01 CI in Nj and 0.33 ± 0.03 CI in jj, *p* < 0.001), while the two genotypes presented similar CI values at P9–10 (0.16 ± 0.008 CI in Nj and 0.16 ± 0.008 CI in jj; *p* > 0.05). Thus, event synchronization in neurons was evident only in the jj samples during the first postnatal week, with a CI that was significantly reduced from P7–8 to P9–10 (*p* < 0.001). Also in the Gunn rat samples, long calcium events were resistant to TTX application (see methods), as shown in the wild-type hippocampal slices or in cultured hippocampal neurons [10].

Our characterization of hyperbilirubinemic Gunn rats showed age-dependent intracellular calcium dysregulations in hippocampal neurons, expressed as longer and synchronized calcium events, both features reminiscent of the alterations induced by exogenous UCB in the wild-type hippocampal slices.

### 3.5. Hippocampal GDPs Are Altered in the Hyperbilirubinemic jj Gunn Rats

In the Nj and jj acute hippocampal slices, we further explored the generation of GDPs by recording glutamatergic pyramidal cells in the CA3 region (top inset in Figure 3D). In this analysis, we directly compared the first vs. the second postnatal week, namely, P5–P6 vs. P8–P9. As shown in Figure 3D–F, at P5–P6, the GDPs were detected in most of both the Nj (N = 9) and jj (N = 11) slices (100% and 75%, respectively; Nj, 2.62 ± 0.61 ms GDP time to half maximum peak and 12,494 ± 5696 mV*ms of area; jj, 1.81 ± 0.18 ms GDP time to half maximum peak and 4122 ± 708 mV*ms of area, *p* > 0.05). However, the GDP frequency was significantly (*p* < 0.05) smaller in the jj rodents than in the Nj ones (from 0.056 ± 0.009 Hz in Nj to 0.027 ± 0.007 Hz in jj; Figure 3D,E).

At P8–P9, the Nj genotype generated GDPs in the majority (69%) of the slices, while only in a minority (25%) of the jj samples (Figure 3C,F; Nj vs. jj *p* < 0.05). The GDP frequency at P8–P9 (Figure 3E) in the Nj slices (N = 13 slices) was 0.042 ± 0.02 Hz, comparable to P5–P6 values, while in the jj samples (N = 8 slices), it was reduced to 0.012 ± 0.011 Hz, but not significantly (*p* = 0.1) probably due to the lower number of observations in the jj slices at this age. P8–P9 GDP time to half maximum peak and areas did not change (Nj, 2.41 ± 0.41 ms GDP time to half maximum peak and 10,776 ± 3980 mV*ms of area; jj, 1.88 ± 0.29 ms GDP time at half maximum peak and 11,832 ± 10,186 mV*ms of area).

In the jj animals, prolonged high bilirubin in the CNS might have affected neuronal integrity, with subsequent changes in hippocampal network activity depending on alterations at the level of glutamatergic pyramidal neurons.

**Figure 4:**
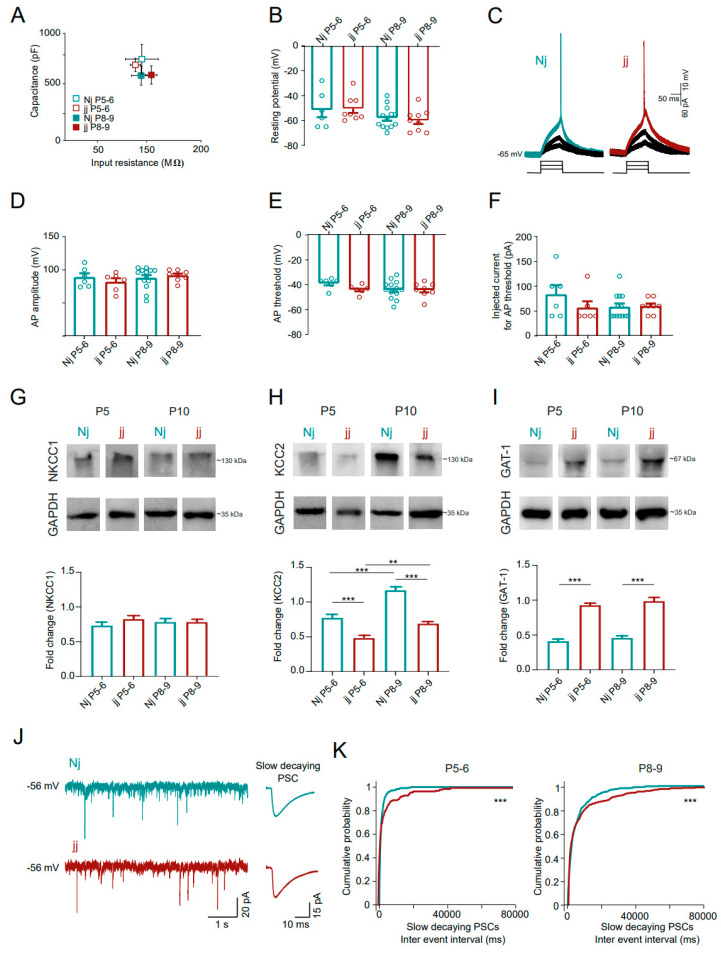
GABAergic markers and PSCs are altered in the hyperbilirubinemic Gunn (jj genotype). (**A**) Plot of cell capacitance (at P5–P6, 113 ± 24 pF for Nj and 112 ± 14 pF for jj; at P8–P9, 103 ± 7 pF for Nj and 127 ± 8 pF for jj) and input resistance (at P5–P6, 760 ± 139 MΩ for Nj and 606 ± 89 MΩ for jj; at P8–P9, 706 ± 61 MΩ for Nj and 611 ± 84 MΩ for jj). (**B**) Bar plot of resting membrane potential (at P5–P6, −51 ± 6 mV for Nj and −50 ± 4 mV for jj; at P8–P9, −58 ± 2 MΩ for Nj and −60 ± 3 mV for jj). At P5–P6, N = 6 cells for Nj and N = 8 cells for jj; at P8–P9, N = 13 cells for Nj and N = 9 cells for jj. (**C**) Traces of induced firing activity. On the bottom, the protocol of stimulation used, with consecutive steps of 10 pA increasing current injections; on the top, corresponding recordings with, in green/red, the sweep in which the threshold for AP was reached and on which AP amplitude was measured. Bar plots of (**D**) AP amplitude (at P5–P6, 89 ± 6 mV for Nj and 82 ± 6 mV for jj; at P8–P9, 88 ± 4 mV for Nj and 91 ± 3 mV for jj), (**E**) AP threshold (at P5–P6, −39 ± 2 mV for Nj and −44 ± 1 mV for jj; at P8–P9, −44 ± 2 mV for Nj and −44 ± 2 mV for jj) and (**F**) injected current to reach the threshold for AP generation (at P5–P6, 83 ± 19 pA for Nj and 57 ± 13 pA for jj; at P8–P9, 58 ± 7 pA for Nj and 60 ± 5 pA for jj). Dots superimposed on the bars are single-cell values. At P5–P6, N = 6 cells for Nj and N = 6 cells for jj; at P8–P9, N = 13 cells for Nj and N = 8 cells for jj. (**G**–**I**) On the top, representative images of Western blot analysis for NKCC1 (**G**), KCC2 (**H**) and GAT-1 (**I**), compared to the housekeeping protein GAPDH, obtained in Nj and jj animals at P5 and P10. On the bottom, plots of the relative fold change in protein expression for the three proteins. Statistical significance by Welch’s *t*-test; N = 6 samples for each condition. (**J**) On the left, representative voltage clamp recordings of spontaneous synaptic activity obtained from Nj and jj slices; on the right, average traces of GABAergic slow-decaying PSCs obtained from the recording on the left. (**K**) Cumulative probability plots of GABAergic slow-decaying PSCs inter-event intervals for animals at P5–6 (**left**) and P8–9 (**right**). At P5–P6, N = 5 cells for Nj and N = 5 cells for jj; at P8–P9, N = 10 cells for Nj and N = 7 cells for jj. Statistical significance by the Kolmogorov–Smirnov test. Statistical differences are reported as ** *p* < 0.01, *** *p* < 0.001.

In the two genotypes, we investigated the electrophysiological properties of CA3 glutamatergic pyramidal neurons. We measured the cell membrane’s passive properties, indicators of neuronal maturation and health [23,24,25], such as the membrane capacitance, the input resistance (Figure 4A), and the resting membrane potential (Figure 4B). No changes were found in these three parameters between the two groups (*p* > 0.05; Figure 4A,B). In both Nj and jj, action potentials (APs), evoked by current pulses (Figure 4C), displayed comparable (*p* > 0.05) amplitude (Figure 4D), threshold (Figure 4E) and injected current to reach the AP threshold (Figure 4F). These results ruled out that the jj slices generated fewer GDPs due to alterations in pyramidal (glutamatergic) cells’ basal passive and/or active membrane properties.

GDPs are generated by glutamate and GABA neurotransmitter interactions, with GABAergic neurons triggering these events due to their depolarizing and excitatory actions in the immature CNS [12,13]. We addressed the presence of alterations due to chronic bilirubin exposure in jj GABAergic neurotransmission and, in particular, in its modulation during development. By Western blot analysis, in the Nj and jj hippocampi, we measured the expression levels of proteins used as GABAergic molecular markers in young rat hippocampi, including Na–K–Cl cotransporter type 1 (NKCC1) and K-Cl cotransporter type 2 (KCC2), both crucial in regulating the developmental change of the chloride gradient across the cell membrane [26], and GABA transporter 1 (GAT-1), which is involved in GABA synaptic re-uptake [27]. Their expression was measured in the whole hippocampus at two temporal points (P5 and P10) and normalized for that of the housekeeping protein GAPDH.

Figure 4G–I show that NKCC1 expression was similar between the Nj and jj groups and, in both groups, there were no changes when comparing P5 and P10 samples (Nj P5: 0.72 ± 0.05 FC, Nj P10: 0.77 ± 0.05 FC; jj P5: 0.82 ± 0.04 FC; jj P10: 0.77 ± 0.04 FC; N = 6 samples each condition, *p* > 0.05; Figure 4G).

Differently, KCC2 expression was significantly smaller in jj with respect to Nj, both at P5 and P10 (Nj P5: 0.77 ± 0.04 FC; Nj P10: 1.17 ± 0.05 FC; jj P5: 0.48 ± 0.03 FC; jj P10: 0.68 ± 0.03 FC; *p* < 0.001 for both the ages; Figure 4H), despite both Nj and jj displaying a significant increase in protein expression from P5 to P10 (*p* < 0.001 and *p* < 0.01, respectively; Figure 4H), in agreement with data reported in the literature [26].

Finally, GAT1 expression was strongly increased in the jj hippocampi when compared to the Nj ones, both at P5 (Nj P5: 0.41 ± 0.03 FC and jj P5: 0.92 ± 0.02 FC; *p* < 0.001) and at P10 (Nj P10: 0.45± 0.03 FC and jj P10: 0.98 ± 0.05 FC; *p* < 0.001; Figure 4I).

To support these findings pointing at a dysregulation of GABAergic neurotransmission in the hyperbilirubinemic Gunn rat, we investigated the GABAergic synaptic inputs received from the CA3 glutamatergic pyramidal neurons. We voltage-clamped single pyramidal cells to record spontaneous synaptic currents. Post-synaptic currents (PSCs) were isolated based on their kinetic properties (see methods), to identify presumed GABAergic slow-decaying (≥15 ms) PSCs (Figure 4J) [28,29]. Slow PSCs (both age groups) displayed longer inter-event intervals in the jj animals when compared to the Nj ones (at P5–P6, 1385 ± 150 ms for Nj and 4359 ± 913 ms for jj; at P8–P9, 5263 ± 347 ms for Nj and 7602 ± 347 ms for jj, *p* < 0.001; Figure 4K). No differences were found in GABAergic slow-decaying PSC amplitudes (at P5–P6, 30 ± 8 pA for Nj and 51 ± 16 pA for jj; at P8–P9, 33 ± 2 pA for Nj and 37 ± 4 pA for jj, *p* > 0.05, Supplementary Figure S2B).

These findings suggest that in the hyperbilirubinemic Gunn rat, GABAergic neuron dysfunctionality may lead to a decreased synaptic input to CA3 glutamatergic pyramidal neurons, ultimately involved in GDP alterations.

## 4. Discussion

The main result of our work is the finding that during the first weeks of postnatal development, elevated bilirubin affects GDP emergence, either transiently increasing their frequency when exogenously applied (acutely) in wild-type slices or severely reducing their occurrence when endogenously increased (chronically) in a hyperbilirubinemic genetic model, i.e., Gunn rats [16]. In hippocampal circuits, alterations due to elevated UCB were coupled in both wild-type and Gunn (jj) slices to prolonged and synchronized intracellular calcium events in pyramidal cells, a hallmark of neuronal stress [30].

In our previous work, exogenous elevated bilirubin in hippocampal cultured neurons induced dose-dependent intracellular calcium dysregulation [10]. In the current work, we adopted acute brain slices, a more complex ex vivo preparation, which, differently from cultured cells, preserves the developmental stage and the cytoarchitecture of the tissue of origin, including the organization in layers and neuronal connectivity [14]. With this approach, we found that elevated bilirubin (exogenously applied in wild-type or endogenously accumulated in jj rats) induced activity-independent long calcium transients, which were synchronized among pyramidal neurons. These events and their synchronization were independent of synaptic activity since they were not affected by TTX. We suggest that other mechanisms, such as the presence of gap junctions [31] or other soluble factors, such as cytokines (CKs), whose release is favored by Bf [32], could promote synchronization, and additional specific studies are needed to clarify this issue.

In previous works, intracellular calcium dysregulation in in vitro neuronal cultures has been ascribed to UCB targeting of the ER, the expression of ER-stress-related proteins and the activation of apoptosis [10,11]. Here, we show, for the first time, that in response to higher bilirubin concentration, similar prolonged and synchronized calcium oscillations were replicated by pyramidal cells in acute slices, but this response was either stronger (exogenous Bf in wild-type) or limited (endogenous hyperbilirubinemia in Gunn rats) to the first postnatal week of hippocampal development. Since, in Gunn rats, hyperbilirubinemia increases after birth and declines in the following weeks [33], the enhanced calcium dysregulations observed during the first postnatal days could be due to higher levels of UCB. However, it is tempting to speculate that the observed effect is related to a narrow CNS developmental window for bilirubin toxicity.

The age-dependency of vulnerability to bilirubin described in our experiments also agrees with previous reports that showed more apoptotic phenomena, glutamate release and neuroinflammation in immature neurons exposed to elevated bilirubin with respect to mature ones [34,35,36]. Such increased sensitivity of younger neurons might emerge due to the combination of several factors, such as the reduced expression of efflux transporters [37], the presence of immature mechanisms of intracellular calcium homeostasis [38] and antioxidant defense [39], the enhanced expression of voltage-gated calcium channels [40] or the higher energy metabolic need [41]. These factors, combined with increased inflammation [42] and age-dependent caspase-3 activation [43], make early postnatal cells more exposed to damaging insults.

During CNS development, the formation and elimination of neural synapses is a dynamic process where appropriate connections are stabilized based on specific patterns of neuronal activity [44]. In the hippocampus, during the first postnatal weeks, GDPs are fundamental for the maturation and consolidation of synapses, and GDPs are also generated by and can be recorded from acute slices [13]. During this critical age, GDPs in wild-type slices transiently increment their frequency in response to bilirubin, an effect in agreement with the enhancement in network activity reported when bilirubin was applied to neurons of the ventral cochlear nucleus at a comparable developmental age [45]. Acute modifications in network activity might involve modulation of glutamatergic [35,46] and/or GABAergic synaptic transmission [45,47] via alteration of presynaptic neurotransmitter release [45,46,47] due to synchronous calcium elevations.

In the hyperbilirubinemic jj genotype, the generation of GDPs was strongly affected during the first and second postnatal weeks when compared to the normobilirubinemic Nj genotype. These changes cannot be directly linked to longer calcium events and potential network hyperexcitability, as in acute WT experiments.

In Gunn rats, where bilirubin elevation is genetically determined, hyperbilirubinemia reaches the maximum peak during the first two postnatal weeks [33], thus matching the time window critical for synaptic maturation, where GDPs are generated in the hippocampus [13]. The premature loss of GDPs in the jj genotype might directly depend on pyramidal cell toxicity, as these neurons are usually involved in GDP generation [48]. However, our electrophysiological results argue against this interpretation, as we did not detect differences in the biophysical properties of pyramidal neurons between Nj and jj slices. Instead, we propose an alternative hypothesis: bilirubin accumulation during early hippocampal development selectively impacts GABAergic interneurons and the GABAergic synaptic inputs into jj pyramidal cells. Supporting this idea, a reduction in GABAergic neuronal density was recently reported in the adult Gunn rat hippocampus [49]. Moreover, in neonatal mice exposed to high bilirubin levels, GABAergic interneurons in the auditory brainstem exhibited selective vulnerability, leading to auditory abnormalities [50].

Based on these findings, we suggest that the altered expression of GABAergic molecular markers observed in the current study might represent a signature of the vulnerability of GABAergic neurotransmission, further supported by our electrophysiological recordings showing weaker GABAergic synaptic inputs to pyramidal cells in the jj genotype. The molecular profile of GABAergic markers in jj is in agreement with this interpretation; indeed, in diverse CNS pathological conditions involving excitotoxic events, KCC2 expression is suppressed [51,52,53]. On the other hand, transgenic mice overexpressing GAT-1 were reported to feature GABAergic synapse decline associated with cognitive impairment and decreased memory retention [54].

## 5. Conclusions

In our study, we propose a role for elevated bilirubin in disrupting the consolidation of neuronal circuitry in the developing hippocampus. During the first postnatal weeks, elevated bilirubin induces age-dependent dysregulation of neuronal intracellular calcium dynamics, a hallmark of neuronal stress, which leads to reduced GDP signaling and alterations in GABAergic markers. These changes likely result in impaired neuronal circuitry consolidation within the hippocampus, contributing to behavioral abnormalities such as reduced object recognition memory observed in adult Gunn rats [55] and the learning impairments reported in patients who experienced perinatal hyperbilirubinemia [5]. Our findings may pave the way for the development of novel therapeutic strategies aimed at regulating intracellular calcium dynamics to prevent or treat bilirubin-induced neurotoxicity.

## Data Availability

The datasets used and/or analyzed during the current study are available from the corresponding author upon reasonable request.

## Supplementary Materials

The following supporting information can be downloaded at https://www.mdpi.com/article/10.3390/cells14030172/s1: Supplementary Figures S1 and S2: “Bf disrupts neuronal calcium oscillations independently of synaptic activity” (.pdf format).

## List of Abbreviations

Bf, bilirubin-free; CI, correlation index; CNS, central nervous system; ER, endoplasmic reticulum; GAT-1, GABA transporter 1; GDPs, giant depolarizing potentials; KCC2, K-Cl cotransporter type 2; NKCC1, Na–K–Cl cotransporter type 1; *p*, postnatal; TTX, tetrodotoxin UCB, unconjugated bilirubin; UGT1A1, uridinediphosphoglucuronate glucuronosyltransferase-1.
