# Supplementary material for: Bilirubin Triggers Calcium Elevations and Dysregulates Giant Depolarizing Potentials During Rat Hippocampus Maturation
Source: Cells. 2025 Jan 23;14(3):172. doi: 10.3390/cells14030172 (PMC11817189; doi:10.3390/cells14030172)
Supplement: Supplementary file 1 [file cells-14-00172-s001.zip › cells-3326369-supplementary.pdf]

cells

MDPI

Additional Material

Bilirubin Triggers Calcium Elevations and Dysregulates Giant Depolarizing Potentials During Rat Hippocampus Maturation

Giada Cellot [1,*,†], Giuseppe Di Mauro [1,†], Chiara Ricci [1], Claudio Tiribelli [2], Cristina Bellarosa [2,*] and Laura Ballerini [1,*]

[1] International School for Advanced Studies (SISSA), Neuroscience Area, Via Bonomea, 265, 34136 Trieste, Italy; gdimauro@sissa.it (G.D.M.); cricci@sissa.it (C.R.)
[2] Fondazione Italiana Fegato ONLUS—Italian Liver Foundation, Bldg Q-AREA Science Park Basovizza, SS14 Km 163.5, 34149 Trieste, Italy; ctliver@fegato.it
* Correspondence: cellot@sissa.it (G.C.); cristina.bellarosa@fegato.it (C.B.); ballerin@sissa.it (L.B.)
† These authors contributed equally to this work.

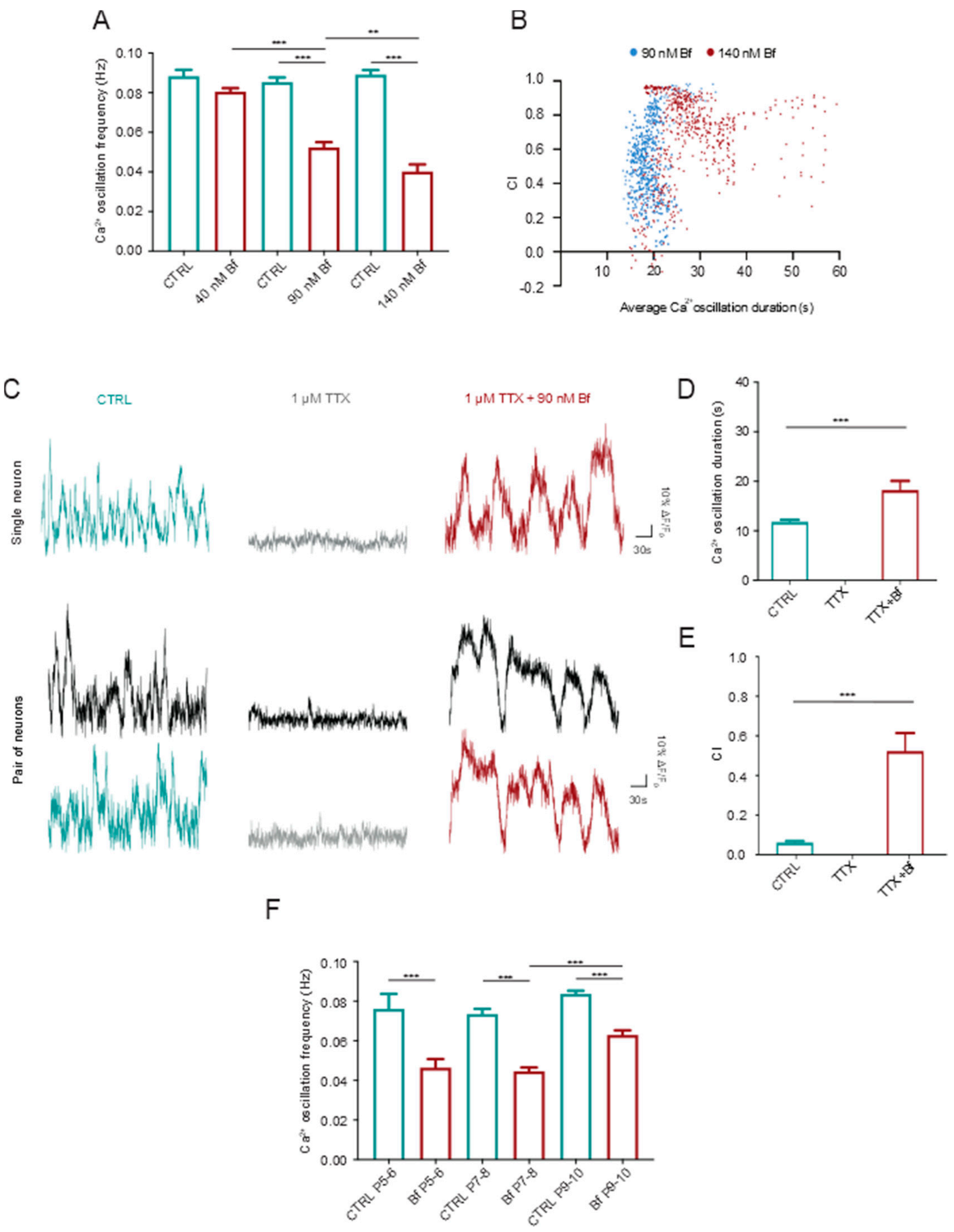

Supplementary Figure S1. Bf disrupts neuronal calcium oscillations independently of synaptic activity. (**A**) Bar plot of Ca^{2+} oscillation frequencies for different Bf doses. A dose of 40 nM Bf did not induce any change in calcium oscillation frequency (control: 0.088 ± 0.003 Hz, 40 nM Bf: 0.080 ± 0.001 Hz, N = 8, $p > 0.05$). Differently, treatments at 90 nM Bf (control: 0.085 ± 0.002 Hz, 90 nM Bf: 0.052 ± 0.002 Hz, N = 8, $p < 0.001$) and 140 nM Bf (control: 0.089 ± 0.002 Hz, 140 nM Bf: 0.040 ± 0.003 Hz, N = 9, $p < 0.001$) induced a statistically significant reduction in oscillation frequency. The comparison between treatments reported statistically significant decreased oscillation frequency between 40 and 90 nM Bf ($p < 0.001$) and 90 and 140 Bf ($p < 0.01$). (**B**) Scatter plot showing the correlation between the average calcium oscillation durations measured in each pair of neurons analyzed in Figure 1E and of the corresponding CI. For 90 nM (in blue) and 140 nM (in red) Bf, Spearman's correlation coefficients were 0.1048 and 0.0540, respectively. (**C**) Representative traces of Ca^{2+} oscillations in CA1 hippocampal neurons before (CTRL, green trace) and after 1 µM TTX (grey trace) or 1 µM TTX + 90 nM Bf (red trace) treatments. On the top row, fluorescent traces from a single neuron are

reported, while on the bottom row, traces from neuronal pairs are shown for the CI analysis. The black traces correspond to the fluorescent profile of another cell in the same optical field. (**D**) Bar plot of spontaneous Ca^{2+} oscillation duration events. The average duration of calcium oscillations was increased from 11.85 ± 0.17 s in the control to 18.85 ± 0.82 s in 1 μM TTX+ 90 nM Bf. (**E**) Bar plot of CI to measure Ca^{2+} oscillation synchronization in pairs of neurons from the same optical field. These were 0.06 ± 0.006 CI in the control and 0.52 ± 0.09 CI in 1 μM TTX + 90 nM Bf. Statistical significance by one-way ANOVA with Tukey's post hoc test; N = 5 samples. Statistical differences are reported as ***$p < 0.001$. (**F**) Bar plot of Ca^{2+} oscillation frequencies at different ages using 90 nM Bf treatment. For all the ages, Ca^{2+} oscillation frequency was decreased in a statistically significant manner after Bf treatment (control P5-6: 0.076 ± 0.007 Hz, Bf P5-6: 0.046 ± 0.004 Hz; control P7-8: 0.073 ± 0.002 Hz, Bf P7-8: 0.044 ± 0.001 Hz; control P9-10: 0.083 ± 0.001 Hz, Bf P9-10: 0.062 ± 0.002 Hz; N = 11 samples and $p < 0.001$ for each condition). No statistically significant differences were observed between Bf P5-6 and Bf P7-8 ($p > 0.05$), but statistically significant differences were observed between P7-8 and P9-10 ($p < 0.001$). Statistical significance was measured by a two-way ANOVA with Sidak's multiple comparison test and reported as ** $p < 0.01$ and *** $p < 0.001$.

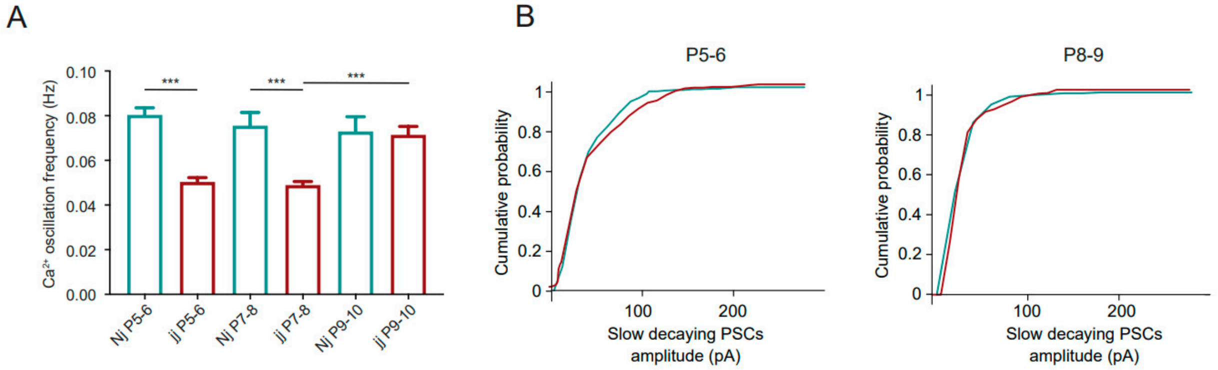

Supplementary Figure S2. Characterization of Ca^{2+} oscillation frequencies and slow-decaying PSC amplitudes in Gunn rats. (**A**) Bar plot of Ca^{2+} oscillation frequencies at different ages in Nj and jj Gunn rats. Differences in Ca^{2+} oscillation frequencies were statistically significant at P5-6 (Nj P5-6: 0.080 ± 0.003 Hz, jj P5-6: 0.050 ± 0.001 Hz, N = 11 and N = 13 samples, respectively, $p < 0.001$) and at P7-8 (Nj P7-8: 0.075 ± 0.005 Hz, jj P5-6: 0.048 ± 0.001 Hz, N = 11 and N = 12, respectively, $p < 0.001$). No differences were observed at P9-10 (Nj P9-10: 0.072 ± 0.006 Hz, jj P9-10: 0.071 ± 0.003 Hz, N = 10 for each genotype, $p > 0.05$). The comparison between jj animals at different ages in terms of Ca^{2+} oscillation frequencies showed no differences between P5-6 and P7-8 ($p > 0.05$), while the increase between P7-8 and P9-10 was statistically significant ($p < 0.001$). Statistical significance was measured by a two-way ANOVA with Sidak's multiple comparison test and reported as ** $p < 0.01$ and *** $p < 0.001$. (**B**) Cumulative probability plots of GABAergic slow-decaying PSC amplitudes for animals at P5-6 (**left**) and P8-9 (**right**). At P5-P6, N = 5 cells for Nj and N = 5 cells for jj; at P8-P9, N = 10 cells for Nj and N = 7 cells for jj. Statistical significance was measured by the Kolmogorov–Smirnov test, $p > 0.05$.
